# Legal Notes for Institutional Workers

**Published:** 1916-09-23

**Authors:** 


### LEGAL NOTES FOR INSTITUTIONAL WORKERS.

### Isolation Hospital Committees.

The House of Lords have affirmed the decision of the Court of Appeal in *Attorney-General* v. *Derbyshire County Council*, 32 T.L.R. 558, holding that where a County Council have by an order established a committee for a hospital district under the Isolation Hospitals Act, 1893, they have power by a subsequent order to alter the constitution of the committee. The circumstances which occasioned the action have already been referred to here, and it is unnecessary to repeat them. Readers will, however, be interested in the conclusion come to by Lord Parmoor, who delivered the principal opinion as to the general effect of the statute mentioned. He thought that the Act did not intend to set up hospital committees of independent status and free from all control of the County Council, but that in many important functions there was a continuing duty on the County Council, and that it was consistent with that duty that the County Council should have power from time to time to alter the constitution of any such committee if differing from them on matters of policy, or if not satisfied with the efficiency of their administration.

### Encouraging Milk Adulteration.

A piece of legislation whose presence on the Statute-book was mainly due to medical opinion and the support of public health authorities has, by a recent decision of the Divisional Court (*Hunt* v. *Richardson*, 32 T.L.R. 560), been practically nullified. We refer to the milk standard set up by the Regulations of the Board of Agriculture, by which it is provided that if a sample of milk is found to contain less than 3 per cent. of milk-fat, it shall be presumed, until the contrary is proved, that the milk is not genuine by reason of the abstraction therefrom of milk-fat, or the addition thereto of water. Hitherto there has been judicial authority for the view that if the milk fell below the standard, owing to the state of the cow's health, the time of milking, or the manner of feeding, then an offence was committed, even though it were shown that the milk was "genuine" in the sense that it was sold as it came from the cow. The decision now given definitely negatives that theory. The facts were that in consequence of heavy rains the grass on which the cows were fed was in a watery condition, and they were giving a much larger quantity of milk than usual. No special steps were taken to counteract the effect of the watery state of the herbage on the quality of the milk. Although the appellant and his servants knew what was taking place, the cows were given green maize, which was even more watery, in order to keep up the quantity of the milk. The analyst's certificate regarding the resulting milk showed that it was deficient in milk-fat to the extent of 9 per cent. The justices found that the deficiency was due to the manner in which the cows were fed, and they convicted the appellant. On appeal it was held by a majority of three judges to two that the milk was genuine, and accordingly the conviction was quashed. The judgment is a victory for the farming and dairying interests, which, ever since the Milk Regulations were made in 1901, have studied and experimented with a view to discrediting the standard percentage. In our view the effect of the decision will be to facilitate the very thing the Legislature wished to guard against, and that is the sale of impoverished milk. The whole object of the law was to protect the consumer; and was not one of the dissenting judges, Mr. Justice Bray, right when he said: "There is no reason why the farmer who feeds his cows in such a way as to reduce the quality of the milk should not be prevented from selling such milk to customers who are ignorant of all the facts; the customer suffers just as much as if the quality were reduced by the addition of water"? But now any liquid which comes from a cow is genuine milk unless it is shown to have been tampered with after leaving the cow, and obviously evidence as to that will practically be unprocurable.

### Health and the Validity of Marriage in the United States.

A branch of medical jurisprudence in which new principle is being slowly yet persistently applied is that relating to marriage. We have already noticed one or two cases even in the British Courts which show appreciation of recent sociological thought. A decision lately issued by one of the Supreme Courts of the United States is notable for its exhaustive consideration of the question of ill-health as an excuse for refusal to fulfil a promise of marriage. That judgment declares that where a man, engaged to marry, subsequently becomes so afflicted by disease that performance of his marriage duties would aggravate his disease and hasten his death, non-performance is excused. This conclusion is arrived at after a review of numerous conflicting authorities. The Court finds generally three cases in which a person who has promised marriage may refuse to perform—(1) when he becomes afflicted with a loathsome disease which may be communicated to the other party or to the children of the marriage; (2) when he becomes afflicted with a fatal and incurable malady and the consummation of marriage would hasten his or her death; and (3) when he or she is stricken with a fatal malady and the evidence makes it reasonably certain that he has but a few days or weeks or months to live. The argument whereby these conclusions are justified has a constant reference to theories of public policy; and it seems to us that we shall not have long to wait till, in America at all events, the Courts will be asked to declare that if one marries, not knowing that the other spouse is afflicted with a disease which soon after develops into a deadly malady, he or she may sue for annulment of the marriage.

